# A Validation of Automatically-Generated Areas-of-Interest in Videos of a Face for Eye-Tracking Research

**DOI:** 10.3389/fpsyg.2018.01367

**Published:** 2018-08-03

**Authors:** Roy S. Hessels, Jeroen S. Benjamins, Tim H. W. Cornelissen, Ignace T. C. Hooge

**Affiliations:** ^1^Experimental Psychology, Helmholtz Institute, Utrecht University, Utrecht, Netherlands; ^2^Developmental Psychology, Utrecht University, Utrecht, Netherlands; ^3^Social, Health and Organisational Psychology, Utrecht University, Utrecht, Netherlands; ^4^Scene Grammar Lab, Department of Cognitive Psychology, Goethe University Frankfurt, Frankfurt, Germany

**Keywords:** eye tracking, Areas of Interest, faces, automatic, videos

## Abstract

When mapping eye-movement behavior to the visual information presented to an observer, Areas of Interest (AOIs) are commonly employed. For static stimuli (screen without moving elements), this requires that one AOI set is constructed for each stimulus, a possibility in most eye-tracker manufacturers' software. For moving stimuli (screens with moving elements), however, it is often a time-consuming process, as AOIs have to be constructed for each video frame. A popular use-case for such moving AOIs is to study gaze behavior to moving faces. Although it is technically possible to construct AOIs automatically, the standard in this field is still manual AOI construction. This is likely due to the fact that automatic AOI-construction methods are (1) technically complex, or (2) not effective enough for empirical research. To aid researchers in this field, we present and validate a method that automatically achieves AOI construction for videos containing a face. The fully-automatic method uses an open-source toolbox for facial landmark detection, and a Voronoi-based AOI-construction method. We compared the position of AOIs obtained using our new method, and the eye-tracking measures derived from it, to a recently published semi-automatic method. The differences between the two methods were negligible. The presented method is therefore both effective (as effective as previous methods), and efficient; no researcher time is needed for AOI construction. The software is freely available from https://osf.io/zgmch/.

## 1. Introduction

In many areas of eye-tracking research, inferences about perception or cognition are drawn by relating eye-movement behavior to the visual stimulus that was presented to the observer. Using this approach, researchers have for example concluded that individuals with social phobia look less at facial features (eyes, nose, mouth) than controls (Horley et al., 2003), that the time to disengage from a centrally presented stimulus decreases with age (Van der Stigchel et al., 2017), and have investigated how information graphics are used (Goldberg and Helfman, 2010). Although data-driven methods exist for this purpose (Caldara and Miellet, 2011), coupling eye-tracking data to the visual stimulus is usually done using so-called Areas of Interest (AOIs) (Holmqvist et al., 2011), especially when there are clear hypotheses to be tested.

There are many ways in which one can construct AOIs, for example by using a grid superimposed on the visual stimulus. Grid-cells may subsequently be combined into AOIs and labeled; e.g., cells X and Y belong to the “nose” AOI in a face (Hunnius and Geuze, 2004). AOIs may also be drawn manually (Chawarska and Shic, 2009)—as is possible with most eye-tracker manufacturer software—or constructed using state-of-the-art computer-assisted techniques (Hessels et al., 2016). When visual stimuli are static (e.g., pictures or schematic drawings), constructing AOIs need only be done once for each stimulus, and this can be achieved with most eye-tracker manufacturer software. However, when visual stimuli are moving (e.g., videos or animated elements on a screen; often referred to as *dynamic* stimuli), constructing AOIs becomes problematic. In this case, AOIs have to be manually constructed for each frame in the video (or each *n*th frame when there is little movement) (Falck-Ytter, 2008; Tenenbaum et al., 2013), which is a time-consuming process. Alternatively, computer software can be programmed to facilitate constructing AOIs. However, this may be beyond the technical skills of many researchers. Here we present software for automatically constructing AOIs in videos containing a face. This fully-automatic technique provides AOI coordinates for the features of a face (left eye, right eye, nose, and mouth) for each frame in the video.

The current state-of-the-art in eye-tracking studies on face-scanning and face-processing exemplifies that a reliable automatic AOI-construction method for videos of a face is needed. There is a large body of literature where videos of faces are shown to participants, to investigate what facial features are looked at (e.g., Võ et al., 2012; Gobel et al., 2015; Pons et al., 2015; Rutherford et al., 2015; Senju et al., 2015; Gobel et al., 2017). In these recent studies, (dynamic) AOIs were manually constructed by the researcher for each individual video, costing valuable researcher time. Additionally, there is a surge in studies investigating gaze behavior during so-called “naturalistic social interactions,” conducted using head-mounted eye-tracking glasses (Ho et al., 2015; Birmingham et al., 2017) and two-way video setups combined with remote eye trackers (Hessels et al., 2017, 2018). Here, each visual stimulus is a unique video containing a moving face (unlike studies where the same videos are used for each participant). In this field, mapping gaze to a video is generally done manually, and it is therefore an even more time-consuming process than manually constructing AOIs for one set of videos. Automatic AOI construction could significantly reduce the time invested by researchers.

In recent work, researchers have attempted to reduce the time spent mapping gaze to a moving stimulus; in this case a video of a face. In their study, Hessels et al. (2017) used a two-way video setup combined with two remote eye trackers to investigate gaze behavior during dyadic interaction (the interaction of two people). A video of the face of one participant was streamed through a live video setup to the other participants (and vice versa). Instead of constructing AOIs on a frame-by-frame basis, they applied a computer-assisted AOI-construction method to the videos. First, the centers of the left eye, right eye, nose and mouth were manually selected in the first frame of the video. Hereafter, the upper portion of the face was selected, in which high-contrast points were detected. The software then tracked these high-contrast points throughout the video. The centers of the left eye, right eye, nose, and mouth were derived from the location of the high-contrast points. In essence, this method is ignorant to the visual stimulus being tracked: it doesn't need a face, but can track high-contrast points in any video. The initial detection of, and the choice for, the facial landmarks (eyes, nose, and mouth) was done by the researcher. The researchers could intervene and manually correct the centers of the left eye, right eye, nose, and mouth if needed. From these centers, AOIs were constructed using the Limited-Radius Voronoi-tessellation method (LRVT) (Hessels et al., 2016). Although Hessels et al. (2017) state that their semi-automatic method drastically reduces time used for AOI construction, it still required action on the researcher's part (1) at the start of the video, (2) whenever there were not enough high-contrast points left to track, (3) whenever there was too much movement such that the face-tracking software lost track, and (4) whenever the researchers deemed the AOI cell centers to have moved off the intended location.

A semi-automatic AOI-construction method (as outlined above) is already an improvement over manual AOI construction methods in terms of the time involved. One may wonder, however, why automatic methods for AOI construction aren't the standard yet in eye-tracking research. With such methods, AOIs could be constructed objectively and in less time than when the researcher constructs the AOIs manually. In fact, face-detection and facial landmark detection methods (detecting locations such as the boundaries of the eyes, nose, and mouth) have been around for a long time (see e.g., Wang et al., 2018, for a comprehensive survey). In many cases, robust detection of facial landmarks can be achieved (see e.g., Baltrušaitis et al., 2013; Li et al., 2018). There are, however, only few examples where such face-detection or facial landmark detection methods have been explicitly applied in eye-tracking research and made available to the public. Examples of face-detection methods used for constructing AOIs are scarce (see e.g., de Beugher et al., 2014; Bennett et al., 2016).

Why are automatic AOI-construction methods not yet commonplace in applied eye-tracking research? We believe that there may be three reasons.

1. The methods that are available may not be reliable enough. In one eye-tracking study in which an automatic AOI-construction method was used (Bennett et al., 2016), the authors stated that “…detection of the eyes was prone to error” and that eyes and mouth were missed in up to 30% of the frames. This is unacceptable for most eye-movement researchers, who are already dealing with eye-tracking data loss due to e.g., difficult participants (e.g., infants; Hessels et al., 2015, school children, or certain patient groups; Birmingham et al., 2017). Empirical researchers are not necessarily interested in automatic methods that are efficient (i.e., run automatically, and don't cost any researcher time), but not as effective (i.e., the AOIs are not adequately constructed) as manual methods, even though these manual methods may be highly inefficient. However, given that the reliability of facial-landmark detection methods is rapidly increasing (Wang et al., 2018) and overall very high (Li et al., 2018), this cannot be the whole story. We believe that other reasons may be more important.
2. The average researcher in experimental psychology may not have the technical expertise to build, adapt or implement a face-detection method for usage with videos of a face.
3. There are no AOI-construction methods available that have been specifically validated against other AOI-construction methods using eye-tracking data.

Our goal in this paper is not to improve or revolutionize existing face-detection or facial landmark detection methods. We see this as the topic of research for computer vision and computer science. Our goal as eye-tracking researchers investigating gaze behavior to faces is to provide an out-of-the-box solution for AOI-construction for videos containing a face, and—most importantly—validate the method. In doing so, we make use of a particularly promising automatic method for detecting facial landmarks: the recently-released OpenFace toolbox (Baltrušaitis et al., 2016). We chose this toolbox, as it is freely available, and appeared to us to work well out-of-the-box. OpenFace excels at facial landmark detection under varying lighting conditions and facial poses (Baltrušaitis et al., 2013). Here we present a fully automatic AOI-construction method using OpenFace (Baltrušaitis et al., 2013, 2016) and the LRVT AOI method (Hessels et al., 2016), and validate it by comparing it to the semi-automatic AOI-construction method previously applied by Hessels et al. (2018) to nearly a hundred videos. We limit the application of the method to videos of frontal recordings of one face, as they are commonly used stimuli in the face-processing literature. By focusing on a small range of video-types, we can optimize on the effectivity of the method. The method presented here combines existing validated techniques (OpenFace for face-detection and facial landmark detection, and LRVT for AOI-research using face stimuli) and is easy to implement by researchers, who might not have the necessary technical skills to develop their own method.

## 2. Methods

### 2.1. Facial landmark detection

Ninety-six videos of participants who were engaged in dyadic interaction through a two-way video setup were taken from a recently published study (Hessels et al., 2018). These videos each contained the frontal view of the face of one person that was in interaction with another person. Overall, there were periods of talking, laughter, making faces, movement of the head, etc. Facial landmark detection was done on these videos using the OpenFace command line binaries, which were retrieved from https://github.com/TadasBaltrusaitis/OpenFace/. These were applied to all videos on a computer running Windows 7.

### 2.2. Area of interest construction

OpenFace detects 68 facial landmarks, which correspond to fixed locations on the face^1^. These facial landmarks were stored in a text file containing horizontal and vertical pixel coordinates for each video frame. As not all 68 landmarks are relevant for the facial AOIs to be constructed, only a subset were used. Table 1 details which specific landmarks were used for deriving the AOI cell centers for the left eye, right eye, nose, and mouth. Using MATLAB R2013a, AOI cell centers were obtained by averaging the coordinates of the OpenFace landmarks for each AOI, and were subsequently stored in a text file with a set of coordinates for each video frame.

**Table 1:** OpenFace landmarks used for deriving Area of Interest cell centers.

**AOI**	**OpenFace landmark numbers**
Left eye	37–42
Right eye	43–48
Nose	31
Mouth	63, 67

**Figure 1:**
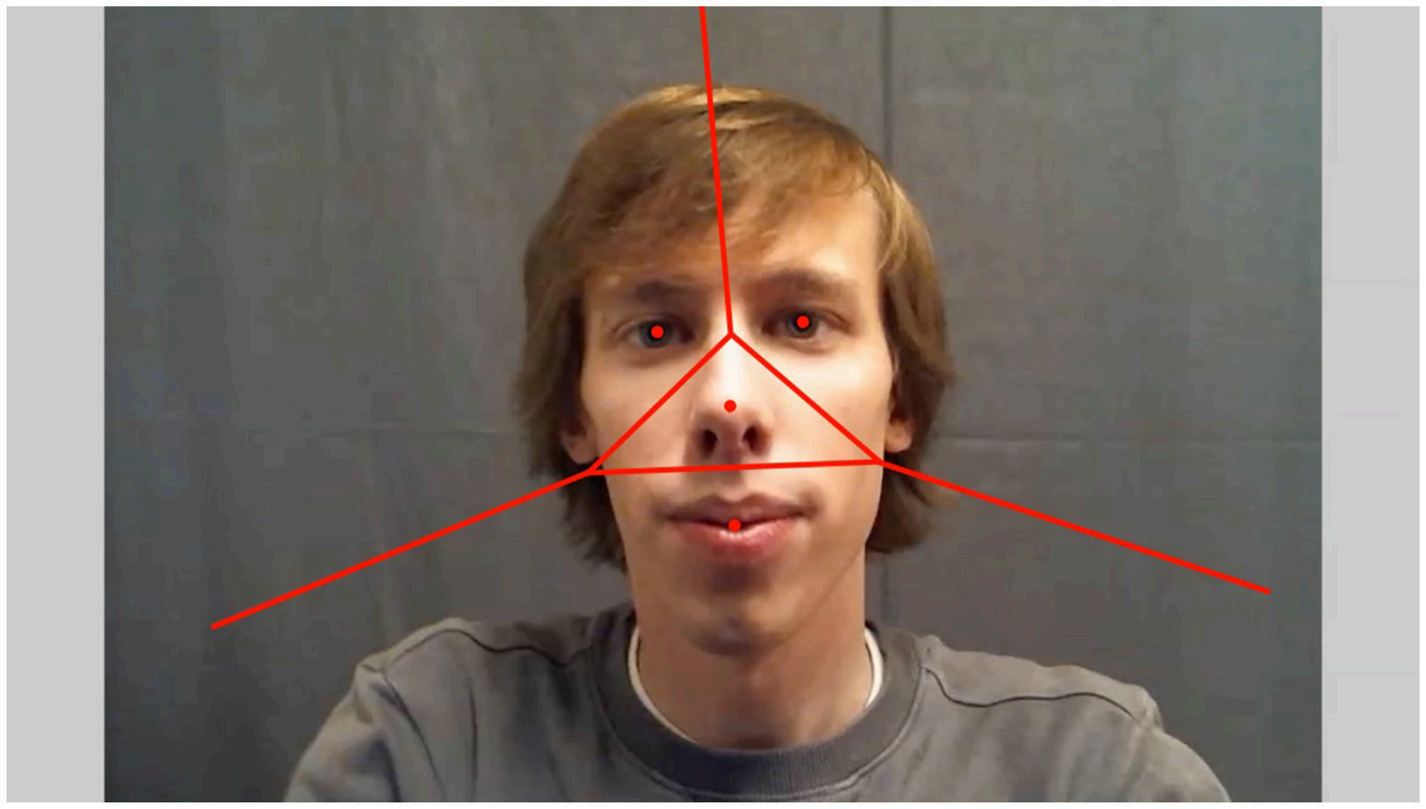
One example frame of a video of the first author recorded with the two-way video setup (Hessels et al., 2017). Area of Interest (AOI) cell centers for the left eye, right eye, nose, and mouth are noted with red dots. Red lines indicate the borders between the AOIs derived from the Voronoi-tessellation method. The ends of these borders are arbitrary; they extend into infinity.

From the AOI cell centers, AOIs were constructed using the LRVT method (Hessels et al., 2016). This method assigns each gaze coordinate to one of four facial features (left eye, right, nose, and mouth) based on which AOI cell center is closest to the gaze coordinate, provided that the distance does not exceed a maximum radius. Any gaze coordinate not assigned to one of the four facial AOIs is assigned to the “non” AOI. Although facial landmarks can be used to construct AOIs in any form desired, the LRVT method was chosen for two reasons. First, previous research has shown that large AOIs (such as LRVT AOIs with large radii) are to be preferred in sparse stimuli (such as faces) (Hessels et al., 2016; Orquin et al., 2016). Second, AOIs in faces created using the LRVT method with a large radius have been shown to be most noise-robust compared with other researcher-defined AOIs (Hessels et al., 2016). The LRVT radius was set to 4°. An example video frame with corresponding AOIs can be seen in Figure 1. As can also be seen, the videos roughly contained the face and upper torso of the participants on a uniform dark-gray background.

## 3. Results

### 3.1. AOI coordinates

In order to validate the automatic AOI-construction method presented here, we compared coordinates of the AOIs as derived from the fully automatic method against a semi-automatic method previously described in the literature. The AOI coordinates for the left eye, right eye, nose and mouth were obtained from the study of Hessels et al. (2018). We assessed (1) whether the AOI coordinates differed systematically between the two AOI-construction methods, and (2) whether the AOI coordinates were more variable over time within one method compared to the other.

**Figure 2:**
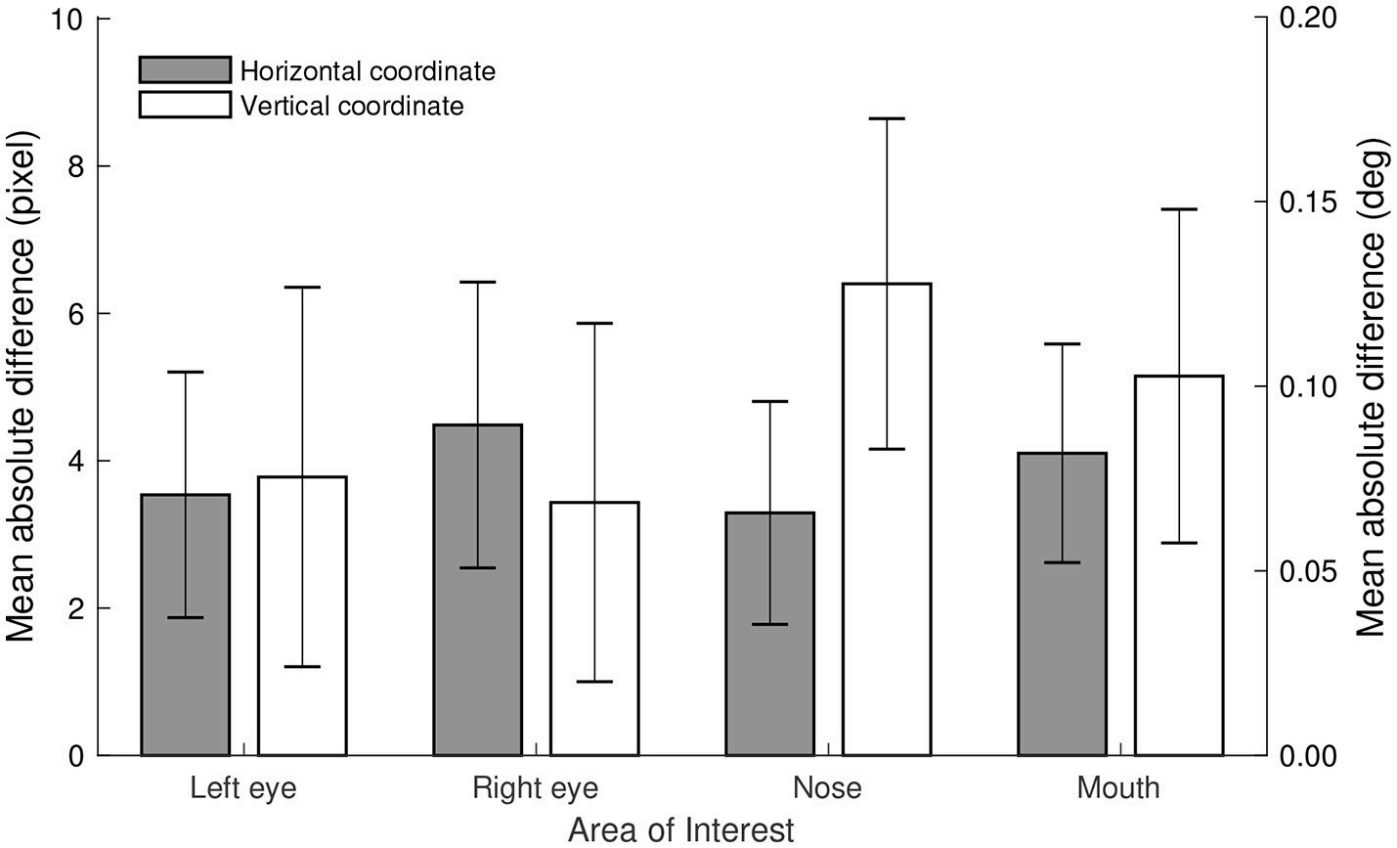
Mean absolute differences in pixels and degrees of visual angle between the coordinates of the Area of Interest cell centers (left eye, right eye, nose, and mouth) as determined by the fully automatic (using OpenFace) and semi-automatic methods. Error bars depict standard deviation calculated across participants.

The systematic difference between the two methods was assessed by calculating the mean absolute difference in AOI positions between the fully automatic and semi-automatic method. As can be seen in Figure 2, the mean difference across the entire video did not exceed 6.5 pixels or 0.13° of visual angle^2^; the largest difference being observed for the vertical coordinate of the nose AOI. These differences are much smaller than the accuracy^3^ values obtained with most eye trackers (around 0.5° of visual angle) (Holmqvist et al., 2011). Moreover, these values are well below the AOI span of 1.9° (the mean distance from each AOI to its closest neighbor Hessels et al., 2016), and merely a fraction of the screen size (22 inch screen of 1680 by 1050 pixels, 47.38 by 29.61 cm, 32.61 by 20.72°). As such, we conclude that the systematic difference in the position of the AOIs between the fully automatic (using OpenFace) and semi-automatic methods is negligible.

**Figure 3:**
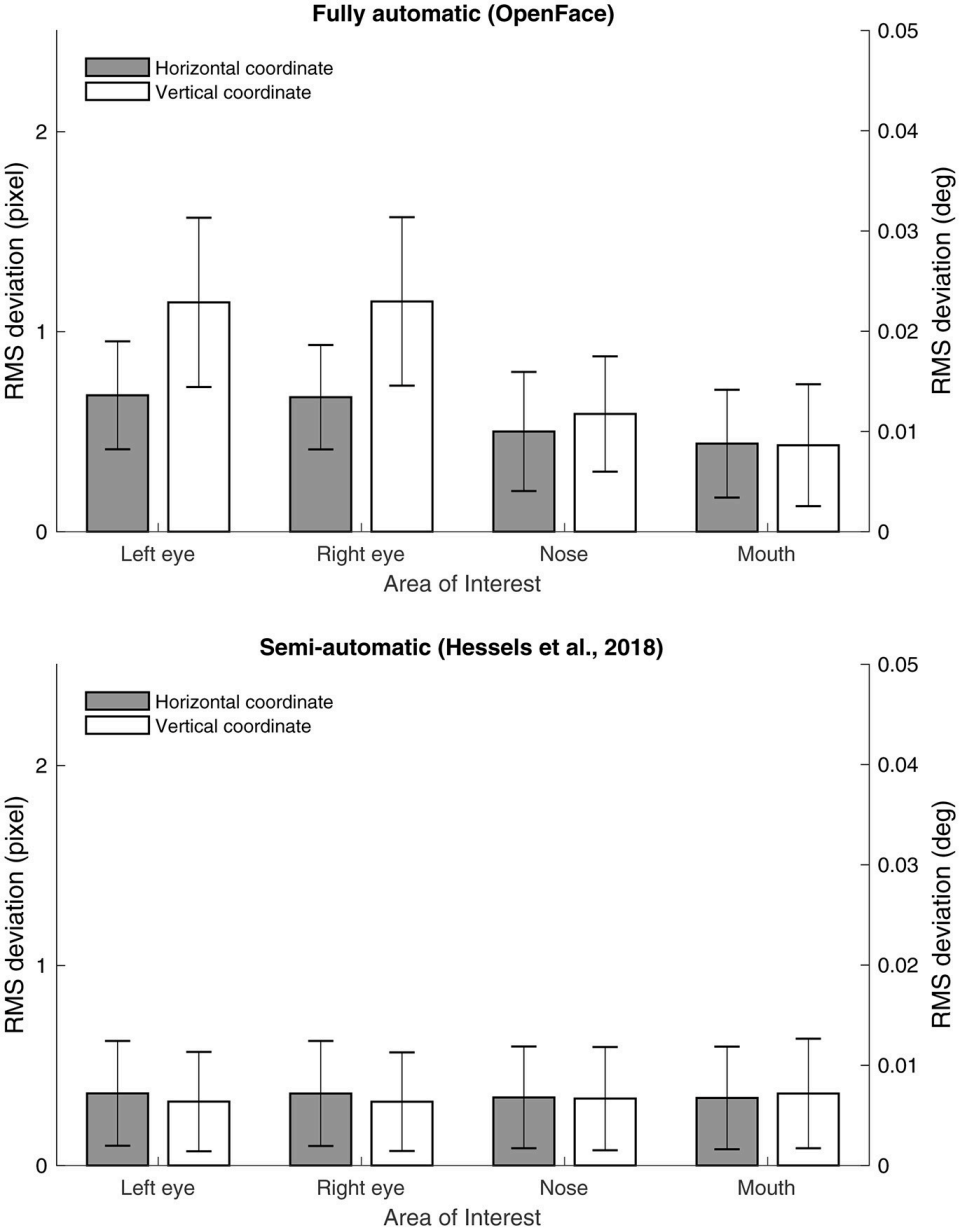
Average root mean square (RMS) deviation of the horizontal and vertical coordinates for the left eye, right eye, nose and mouth as determined by the fully automatic method **(Top)**, and the semi-automatic method **(Bottom)**. Error bars depict standard deviation calculated across participants.

While the systematic difference between the AOI coordinates as determined by the two methods was small, it may be that the AOI coordinates of one method are more variable over time. This can occur, for example, if the facial landmark detection is unstable over time. To examine whether this was the case, we calculated the root mean square (RMS) deviation of the horizontal and vertical coordinates for the cell centers of the four AOIs (left eye, right eye, nose, and mouth) for each method. If the fully automatic method is less stable over time, this should be visible as a larger RMS deviation in the AOI coordinates. As can be seen in Figure 3, the RMS values were all below 2 pixels (or 0.04° of visual angle), well below the optimal accuracy in most eye trackers as well as the AOI span. However, the RMS values were somewhat higher for the fully automatic than for the semi-automatic method. The reason for this is at least 2-fold: (1) the semi-automatic method derives AOI position from a large number of high-contrast points, making it less susceptible to changes in pixel intensities at the location of the facial feature (2) the semi-automatic method does not continue tracking the face when there is too much movement, whereas the fully automatic method does.

**Figure 4:**
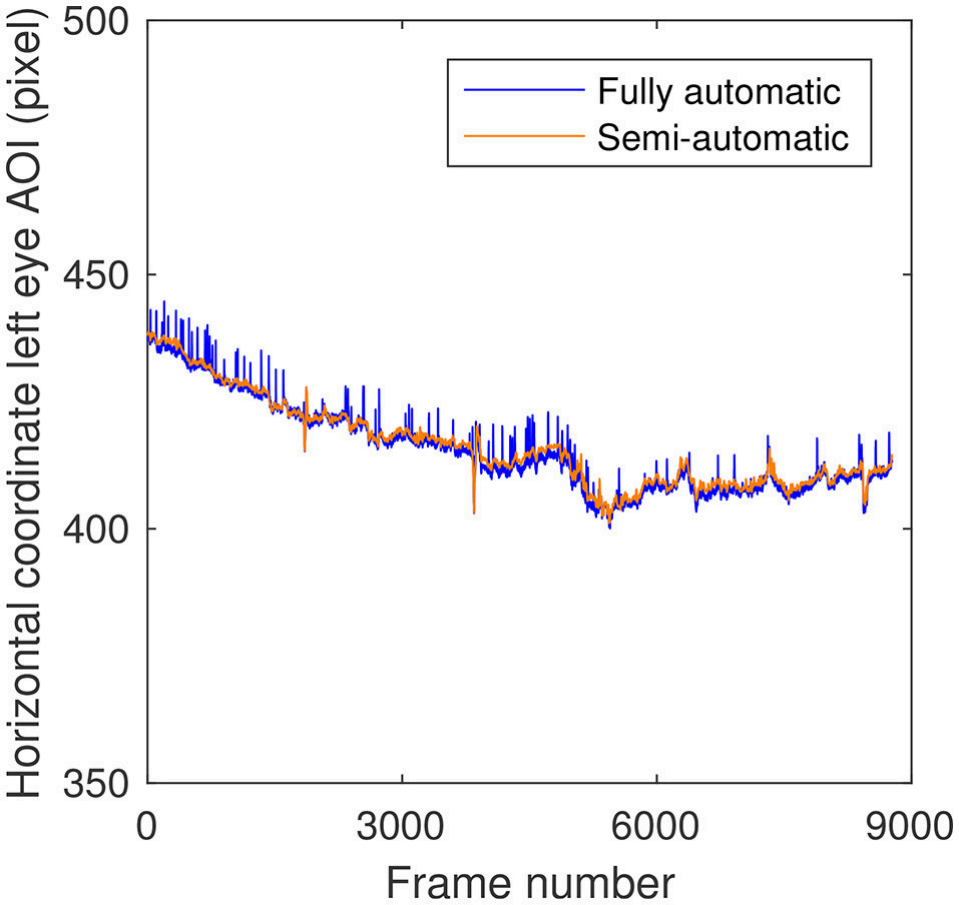
Horizontal coordinate of the left eye Area of Interest (AOI) for the fully automatic (using OpenFace) and semi-automatic AOI-construction methods as a function of video frame number for one example video.

Upon closer examination, another likely factor contributing to higher RMS values for the fully-automatic method was uncovered. Figure 4 depicts the horizontal coordinate of the left eye AOI as acquired by the fully automatic and semi-automatic methods. Although the two signals almost completely overlap, there are several instances in the fully-automatic method where there is a small upward spike in the horizontal coordinate. These spikes were observed for the horizontal and vertical coordinates of the two eye AOIs, yet not for the nose and mouth. On all occasions, the spikes were downward and inward (toward the center) in the video frame. Upon inspection of the videos, these spikes seemed to correspond to the eye blinks made by the participants. In order to ascertain the size of these spikes, the maximum absolute difference in the coordinates of the left eye and right eye between the two methods were determined for each participant and subsequently averaged across participants. Note that the value per participant corresponds to a single sample, not an average across samples. The values thus obtained were below 20 pixels or 0.4 degrees of visual angle. The largest value obtained for one participant overall was just below 45 pixels or 0.9 degrees of visual angle. It should be noted that this value includes any systematic difference in AOI coordinates between the two methods as well—the difference may not solely be caused by the spike due to the eye blink.

### 3.2. Eye-tracking measures

Although both the systematic and variable differences between the fully-automatic method and the semi-automatic method were below the optimal accuracy values of most eye trackers (i.e., AOI position is more precise and accurate than the eye-tracking signal), the negligibility of the differences can best be shown by comparing the eye-tracking measures obtained using both methods. We compared total dwell times, average dwell times, and number of dwells to the left eye, right eye, eyes (left and right eye AOIs combined), nose, mouth, and non AOI as derived using the two methods. Moreover, the original study (Hessels et al., 2018) featured two participants looking at a live stream of each other. As such, we could also compare measures on the frequency, average duration, and total duration of the paired gaze states that participants could engage in. These paired gaze states corresponded to periods when participants were looking at each other's eyes simultaneously (two-way eye gaze), when only one was looking at the eyes while the other was not (one-way eye gaze), and when both were not looking at the eyes (no eye gaze). If the method used to determine AOI cell centers affects eye-tracking measures, measures of paired gaze states should be most affected, as they are derived from a combination of AOI data of two participants. As visible from Table 2, differences between the eye-tracking measures as derived from both methods were small (≤ 2.6 %). This indicates that eye-tracking measures were not affected by which AOI method was used (either the fully-automatic or the semi-automatic AOI method). Hence, the differences in the coordinates of the AOI cell centers between the two methods are evidently negligible.

**Table 2:** Total dwell time (TDT) and total time of paired gaze states as derived from the fully-automatic and semi-automatic methods, and the difference between the two methods in seconds and percentage.

**Measure**	**Full automatic**	**Semi-automatic**	**Difference (s)**	**Absolute difference (%)**
TDT left eye (s)	69.79 (*sd* = 47.05)	70.81 (*sd* = 47.6)	−1.021 (*sd* = 4.142)	1.4
TDT right eye (s)	65.95 (*sd* = 51.89)	64.46 (*sd* = 51.87)	1.498 (*sd* = 3.375)	2.3
TDT eyes (s)	135.7 (*sd* = 70.22)	135.3 (*sd* = 71.27)	0.4764 (*sd* = 5.562)	0.4
TDT nose (s)	52.17 (*sd* = 41.21)	53.56 (*sd* = 42.24)	−1.397 (*sd* = 5.611)	2.6
TDT mouth (s)	33.99 (*sd* = 37.69)	33.39 (*sd* = 37.09)	0.5945 (*sd* = 2.863)	1.8
TDT non (s)	20.12 (*sd* = 30.88)	20.13 (*sd* = 30.85)	−0.00525 (*sd* = 0.6876)	0.0
Total time two−way (s)	71.22 (*sd* = 51.24)	71.16 (*sd* = 52.22)	0.05629 (*sd* = 4.149)	0.1
Total time one−way (s)	133.8 (*sd* = 37.69)	133.3 (*sd* = 38.27)	0.5348 (*sd* = 4.139)	0.4
Total time no eye gaze (s)	67.8 (*sd* = 52.05)	68.92 (*sd* = 53.41)	−1.122 (*sd* = 4.497)	1.6

*Total dwell time was calculated for the left eye, right eye, eyes (both eye AOIs combined), nose, mouth, and non AOIs. Total duration was calculated for the two-way eye gaze, one-way eye gaze, and no eye gaze paired gaze states. Percentage absolute difference is calculated as the absolute difference between the methods in seconds divided by the value for the semi-automatic method*.

## 4. Discussion

Here we have presented and validated an automatic AOI-construction technique based on a combination of recent computer vision and AOI techniques that automatically achieves AOI construction for videos of a face. This technique consists of OpenFace (Baltrušaitis et al., 2013, 2016) for facial landmark detection and the Limited-Radius Voronoi-tessellation AOI method (Hessels et al., 2016). The technique was validated against semi-automatic AOI construction in 96 videos of videos of faces acquired from a study on gaze behavior in social interaction using a two-way video setup (Hessels et al., 2018). The systematic difference between the AOI coordinates as determined by the semi-automatic, and the fully automatic (using OpenFace) methods was far below the average accuracy of modern eye trackers (Holmqvist et al., 2011). This was also the case for the variable difference assessed using the root mean square (RMS) deviation of the AOI positions as derived from both methods. Moreover, eye-tracking measures as derived from the two methods differed ≤ 2.6% from each other. Therefore, the systematic differences between the two methods are negligible.

Upon inspection of the AOI coordinates of the fully automatic method, we found small spikes in the AOI coordinates when participants blinked. When a participant blinked, the AOI coordinates of the eyes moved slightly downward and inward (i.e., toward the nose). Although the magnitude of these spikes was on average below the accuracy achieved by most eye trackers, we will briefly consider the consequences for data analysis. If the eye blink of a person in the video is registered as a change in the coordinate of the eye AOI cell center, it may be that the gaze position of an observer who looks around the border between two AOIs (e.g., the left eye and the nose) is briefly assigned to a different AOI. One might argue that a moving, talking and blinking face is inherently dynamic, which means that the AOIs move in the video, both together (e.g., a translation of the face) or with respect to each other (e.g., when different facial expressions are made). However, if one considers these spikes in AOI coordinate due to blinks to be a problem, it is possible to detect them and filter them from the signal. Here, we found no evidence that eye-tracking measures were affected by which AOI method was used. To sum, the fully automatic AOI-construction method presented here produces AOI coordinates as reliably as previously used semi-automatic approaches. As it does not need researcher input, however, it greatly improves over manual or semi-automatic AOI-construction methods.

As stated in the introduction, there is a large interest in gaze behavior to facial features, both to video and during “naturalistic social interactions.” Mapping eye-tracking data to the visual stimulus requires time-intensive manual coding, which calls for an automatic method. Even though automatic techniques have previously been proposed (e.g., Bennett et al., 2016), the de facto standard for videos of a face in eye-tracking research is still manual AOI-construction. As we have stated, this might be due to the fact the available methods for such purposes specifically are not reliable enough. Moreover, it may be that researchers in experimental psychology do not have the technical expertise to build or implement existing face-detection and/or facial landmark detection methods for use in eye-tracking research. Finally, no automatic AOI-methods have been extensively validated against other AOI-construction methods using eye-tracking data. Here we have presented a first fully automatic AOI-construction method for videos containing a face in eye-tracking research. We have validated the method and shown that is more *efficient* and at least as *effective* as a previously published semi-automatic method. We therefore believe it has a high potential utility in the applied eye-tracking fields of face scanning, face processing, etc.

We have taken a state-of-the-art toolbox for facial-landmark detection (OpenFace) to construct AOIs for one specific problem in eye-movement research, namely investigating one's gaze behavior when looking at the moving and deforming face of another. OpenFace is, however, capable of tracking more than one face in a video, as are other techniques in the literature (e.g., Farfade et al., 2015). In the future, automatic AOI-construction methods that are built upon those techniques can be validated for use as an automatic AOI-construction methods using eye-tracking data. Such validation studies are important to ascertain the robustness and reliability of automatic AOI-construction methods and may be a great push forward for the field of face perception. There is much to be gained by incorporating computer vision/computer science techniques in applied eye-tracking research. Using such a technique we have tackled one specific problem.

## 5. Conclusions

In order to map eye-movement behavior to the visual information presented to an observer, Areas of Interest (AOIs) are commonly employed. Constructing AOIs for static stimuli requires that one AOI set is constructed for each individual stimulus, and this is possible with most eye-tracker manufacturer software. For moving stimuli, however, it is often a time-consuming process, as AOIs have to be constructed for each frame of the video. We've validated a fully automatic AOI-construction method for videos of faces based on OpenFace facial landmark detection and Voronoi-tessellation. The difference between the AOI coordinates derived from this method and a semi-automatic method was negligible, as were the eye-tracking measures derived from them. This means that the method is at least as effective as manual or semi-automatic AOI construction. Moreover, as the AOI-construction method is fully automatic it is highly efficient, and can save valuable researcher time. Given the effectivity and efficiency of the present method, we believe it could become the new standard in applied eye-tracking fields where videos of faces are used. The software is freely available from https://osf.io/zgmch/. Technical details, requirements and instructions are given in Supplementary Data Sheet 1.

## Author contributions

RH and IH conceived the study. RH, JB, and TC wrote the MATLAB code implementing the AOI-construction method on the pre-existing OpenFace toolbox. RH performed the data analysis and drafted the manuscript. All authors commented on and helped finalize the manuscript.

### Conflict of interest statement

The authors declare that the research was conducted in the absence of any commercial or financial relationships that could be construed as a potential conflict of interest.

## Footnotes

^1^see https://github.com/TadasBaltrusaitis/OpenFace/wiki/Output-Format for the location of each landmark.

^2^Visual angles are reported under the assumption that participants remained at 81 cm from the screen and in the center of the camera image.

^3^Accuracy refers to the systematic offset between the gaze position as reported by the eye tracker, and the actual (or instructed) gaze position of the participant.
