# Supplementary material for: A Validation of Automatically-Generated Areas-of-Interest in Videos of a Face for Eye-Tracking Research
Source: Front Psychol. 2018 Aug 3;9:1367. doi: 10.3389/fpsyg.2018.01367 (PMC6085555; doi:10.3389/fpsyg.2018.01367)
Supplement: Supplementary file 1 [file Data_Sheet_1.pdf]

Supplementary Data Sheet 1

System requirements and copyright

The software presented here was built using MATLAB R2013a, the PsychToolbox version 3.0.13 (Brainard, 1997) and pre-compiled OpenFace binaries for Windows. The software was tested on Windows 7 Enterprise SP1 64-bit, but is expected to function with most recent Windows and MATLAB distributions. The software is available from https://osf.io/zgmch/. Newer versions of the OpenFace binaries may be available from https://github.com/TadasBaltrusaitis/OpenFace/releases.

When using this software, please cite the following works: the OpenFace toolbox (Baltrušaitis, Robinson, & Morency, 2016), the facial landmark detection submodule (Baltrušaitis, Robinson, & Morency, 2013), the limited-radius Voronoi-tessellation method (Hessels, Kemner, van den Boomen, & Hooge, 2016), and the present work for the full implementation.

Software overview and instructions

The software consists of one main MATLAB script (*getOpenFaceAOIs.m*) and several dependencies (a folder called *Functions* and the *FeatureExtraction.exe* executable). When *getOpenFaceAOIs.m* is run, the following steps are executed:

1. The number of videos in the folder *videos* is determined. For each video file, steps 2–7 are executed. One example video (*testvideo.avi*) is provided with the software, and the filenames stated below are therefore in reference to this example video. Note that this video is not one of the 96 analysed for validation of the method. The testvideo is more dynamic (in terms of movement), and at times shows the limits of what may reliably be tracked.

2. The video is passed to the landmark detection module of the OpenFace toolbox. This results in the following files being saved in the *output* folder: *testvideotrack.avi*, which depicts the tracking result from the OpenFace toolbox,

and *testvideo_raw.txt*, which contains horizontal and vertical coordinates for all the 68 landmarks detected by the OpenFace toolbox for each frame of the video.

3. The relevant landmarks for the left eye, right eye, nose, and mouth AOIs are extracted from the *testvideo_raw.txt* file, and horizontal and vertical coordinates for these AOI centers are saved in *testvideo.txt* in the *output* folder. Note that the left eye corresponds to the eye that is left in the video, not to the eye that may be considered left from the perspective of the person (and vice versa for the right eye). AOI cell centers are scaled to the size of the video as it was presented on the screen during the experiment, as the eye-tracking data are expected to be in these coordinates. The value for the size of the video during the experiment may be changed in the setting *video.showSize*.

4. A movie is exported to the *output* folder with the name *testvideo.mp4*. This video depicts the locations of the AOI cell centers for later verification.

5. For each video, a corresponding file with eye-tracking data is loaded from the folder *eyetracking data*; in this case *testvideo.txt*. This file contains four columns: time in seconds, horizontal gaze position, vertical gaze position, and a frame number. The frame number corresponds to a frame in the original *testvideo.avi*. Note that gaze positions are given in coordinates of the movie as it was shown on the screen (with the origin corresponding to the top left corner of the video).

6. A new file (*testvideo_allData.txt*) is created in the *output* folder in which the eye-tracking data and the AOI cell centers are combined.

7. For each sample in the eye-tracking data, the AOI For each sample, the AOI is determined based on the limited-radius Voronoi-tessellation method. In short, this method assigns to each sample the AOI of which the cell center is closest to the gaze position, provided that the distance from AOI cell center to the gaze position does not exceed the radius given in the *radius* setting (given in pixels). When the distance from the gaze position to the closest AOI cell center does exceed the

radius, the value “NON” is added as the AOI. When no gaze position is reported for a sample in the eye-tracking data, the value “NOETDATA” is assigned as the AOI. When a gaze position is reported, but no coordinates for the AOI cell centers are given, the value “NOAOIDATA” is assigned as the AOI. The result of this method is then saved to *testvideo_allDataAOI.txt* in the *output* folder.

The final file that is produced for each video (*testvideo_allDataAOI.txt*) may further be processed in a number of ways in order to extract AOI-based eye-tracking measures. For example, one can downsample the eye-tracking data to the frequency of the video before calculating sample-based dwells. Alternatively, fixations can be detected from the raw eye-tracking data, and fixation-based dwells determined. Hereafter, number of dwells, (total) dwell times for the facial AOIs and transitions between dwells can be calculated. The reader is referred to Holmqvist, Nyström, Andersson, Dewhurst, Jarodzka, & van de Weijer (2011) for more information on calculating these specific measures.

References

Baltrušaitis, T., Robinson, P., & Morency, L.-P. Constrained Local Neural Fields for Robust Facial Landmark Detection in the Wild. In *2013 IEEE International Conference on Computer Vision Workshops (ICCVW)*, pages 354–361. IEEE, (2013).

Baltrušaitis, T., Robinson, P., & Morency, L.-P. OpenFace: an open source facial behavior analysis toolkit. In *IEEE Winter Conference on Applications of Computer Vision*, pages 1–10, (2016).

Brainard, D. H. (1997). The Psychophysics Toolbox. *Spatial Vision*, 10(4):433–436.

Hessels, R. S., Kemner, C., van den Boomen, C., & Hooge, I. T. C. (2016). The area-of-interest problem in eyetracking research: A noise-robust solution for face and sparse stimuli. *Behavior Research Methods*, 48(4):1694–1712.

Holmqvist, K., Nyström, M., Andersson, R., Dewhurst, R., Jarodzka, H., & van de Weijer, J. (2011). *Eye tracking: A comprehensive guide to methods and measures.* Oxford University Press.
